# Accuracy of working length determination using endodontic motors with integrated electronic apex locators: an *in vitro* study

**DOI:** 10.3389/froh.2026.1885521

**Published:** 2026-07-23

**Authors:** Huynh-Anh Bui, Minh-Anh Bui, Thi-Thuy-Trang Huynh, Hoang-Lan-Anh Le, Phuong-Doan Phan, Nguyen-Tra-Mi Le, Thuan-Loc Tran, Thi-Bich-Van Tran, Quoc-Viet Lam, Van-Khoa Pham, Trieu-Khang Pham, Huynh-Thuy-Anh Bui, Cong-Minh Nguyen, An-Tran Pham, Tran-Lan-Khue Pham, Quynh-Anh Lam

**Affiliations:** 1 Faculty of Dentistry, University of Medicine and Pharmacy at Ho Chi Minh City, Ho Chi Minh City, Vietnam; 2 Faculty of Operative Dentistry and Endodontics, National Hospital of Odonto-Stomatology, Ho Chi Minh City, Vietnam; 3 Faculty of Odonto-Stomatology, University of Health Sciences, Vietnam National University Ho Chi Minh City, Ho Chi Minh City, Vietnam

**Keywords:** auto apical reverse, electronic apex locator, endodontic motor, integrated apex locator, root canal length, working length

## Abstract

The aim of the present study was to evaluate the accuracy of endodontic motors with an integrated electronic apex locator (EAL) for determining endodontic working length under *in vitro* conditions. A total of twenty-six extracted human mandibular single-canal premolars were included and scanned via cone-beam computed tomography (CBCT), three-dimensionally reconstructed, and then allocated equally into two device groups (*n* = 13 each). The teeth were accessed and a glide path was prepared with a manual K-file before the canals were prepared with a continuous single-file system (One Curve, Micro-Mega, Besançon, France) driven by one of two integrated endodontic motor/EAL systems (Connect Drive, VDW; and E-Connect S, Eighteeth) until the auto-apical-reverse (“apex”) signal was displayed. The teeth were scanned by CBCT a second time after preparation, and 3D reconstruction was performed to determine the post-preparation endpoint. Subtraction of the pre- and post-preparation images was used to evaluate the accuracy of the working length relative to the directly measured actual length. The data were analysed using Bland–Altman agreement analysis (mean bias and 95% limits of agreement) in MedCalc, with between-device comparison performed using a test appropriate for the independent two-group design; significance was set at 0.05. There was no significant difference in working length between the two integrated systems. Under the present *in vitro* conditions, neither integrated motor/EAL system produced measurements identical to the reference length, although the deviations were small: the mean bias of the auto-apical-reverse endpoints relative to the actual length ranged from approximately 0.13 to 0.26 mm, remaining within the ±0.5 mm clinical tolerance, and the largest single deviation recorded was 0.79 mm short of the major apical foramen. The working length should therefore be confirmed using complementary methods rather than relying solely on the auto-reverse endpoint.

## Introduction

The working length is one of the most important determinants for the success of root canal therapy (1–5). All endodontic procedures require confinement inside the root canal space to ensure that the periapical region is not further irritated (4, 6).

There are many modalities for determining the endodontic working length, from paper-point measurement to the electronic apex locator and combinations of these with digital radiographs, including periapical imaging and cone-beam computed tomography (CBCT) (4, 6, 7).

Periapical digital radiographs are not available in all circumstances, especially when the apical foramen is on the buccal or lingual root surface because of the vestibulo-palatal direction of the x-ray beam. The magnification amplitude also differs across locations on a digital image, which can result in incorrect measurements of root canal length (2, 4). With the advancement of software, the accuracy of determining the working length on digital radiographs has considerably improved (6, 7).

Cone-beam computed tomography (CBCT) is a contemporary imaging modality in dentistry (8). Distance measurement using CBCT software is a reliable, repeatable, and accurate tool in implantology, periodontology, and endodontics (3). Several previous studies have shown that root canal length measurements are reliable and precise, although the length measured by this modality may differ from the actual length (1–4). Drawbacks of CBCT for root canal length include the need for an available scan, the appropriateness of slice thickness, difficulty in selecting proper sectional slices, and image clarity for landmark determination (1–4). Radiation exposure is a concern whenever CBCT is used routinely because of the associated risk-benefit balance (8).

The electronic apex locator is a reliable device for root canal length determination and has become a standard of care in endodontic therapy. Multiple mechanisms underlie apex-locator performance, and this technology has been continuously improved (1, 3, 5).

The root canal length measured by a conventional electronic apex locator is typically static. However, the canal length changes during preparation, especially in severely curved canals. Root canal length is therefore a dynamic concept that should be monitored throughout endodontic preparation (9).

The endodontic motor with an integrated electronic apex locator is a modern modality intended to fulfil the requirement of dynamic working length determination (6). This combined system controls the working length during instrumentation, ensuring that the instrument works within a permitted range. Depending on the selected option, the preparation instrument can be automatically reversed (“auto apical reverse”) or stopped when it reaches the apical foramen. If the operator relies entirely on the integrated system, an over-extended foramen cannot be repaired when the system is less precise than expected (1–3, 5, 10). To date, the combination of periapical radiographs and electronic apex locators has been regarded as one of the most accurate modalities for working length estimation (2, 5). By contrast, comparatively few studies have evaluated the accuracy of integrated endodontic motor/EAL systems in the dynamic situation (6, 11), and existing reports differ in devices, reference standards, and analytic methods. The specific gap addressed here is the accuracy of two contemporary integrated motor/EAL systems (Connect Drive and E-Connect S) operating in auto-apical-reverse mode, evaluated against a directly measured reference length using a CBCT-based three-dimensional subtraction protocol — a combination not previously reported.

The aim of the present study was to evaluate the accuracy of the root canal length determined by an endodontic motor-integrated electronic apex locator in extracted human premolars. The first null hypothesis was that there would be no difference between the root canal length measured by the integrated electronic apex locator and the actual length. The second null hypothesis was that there would be no difference between the two experimental endodontic motors in the deviation between measured length and actual length.

## Materials and methods

### Ethical approval and specimen source

This study was approved by the Research Ethics Committee of the University of Medicine and Pharmacy at Ho Chi Minh City, Vietnam (approval number 296/HĐĐĐ-ĐHYD, 10 March 2022). The teeth were mandibular premolars extracted as part of routine orthodontic treatment and were collected anonymously after extraction. Written informed consent for the use of the extracted teeth for research was obtained from the patients or, for minors, from their legal guardians, in accordance with national legislation and institutional requirements. No potentially identifiable images or personal data are presented; consequently, separate consent for the publication of identifying information was not required. All methods were performed in accordance with the relevant guidelines and regulations.

### Sample size estimation

The sample size was calculated using G*Power version 3.1.9.6 (Universität Kiel, Germany) for the primary within-device comparison of the post-preparation length against the directly measured actual length. A matched-pairs analysis was specified with an effect size of 0.75 and power of 0.80, yielding 13 canals per device group (26 teeth in total). We acknowledge that an alpha of 0.10 was used for the *a priori* sample-size estimation to limit the type II error in this exploratory *in vitro* design, whereas inferential analyses were performed at alpha = 0.05; this discrepancy and the modest sample size are stated explicitly as limitations, and the study was powered for the within-device paired comparison rather than for the between-device contrast.

### Specimen selection and inclusion criteria

A total of twenty-six human mandibular single-canal premolars extracted from orthodontic patients were included. Single-canal anatomy was confirmed on a preoperative periapical radiograph and under a dental operating microscope. Teeth were excluded if they showed external or internal defects, cracks, caries extending to the canal, immature or resorbed apices, calcification, severe curvature, multiple foramina, or previous endodontic treatment; any tooth with such findings was discarded. CBCT scans with a voxel size of 0.075 mm, 90 kVp, 8 mA, field of view (FOV) of 50 × 50 mm were acquired for each tooth before preparation, with the crown embedded in putty silicone in a plastic round bowl. The operator performing the measurements was blinded to group allocation and to the actual length.

### Reference (actual) length determination

The teeth were accessed, and a glide path was prepared with a manual K-file so that the entire patent canal was accessible to the apical foramen. The actual length was then measured with a digital ruler from the occlusal reference point to the tip of a #10 ISO K-file (Dentsply Sirona, Ballaigues, Switzerland) after the file tip was observed under a dental operating microscope at ×10 magnification at the level of the major apical foramen (defined as the point at which the file tip first became visible at the major foramen).

### Instrumentation with the integrated motor/EAL systems

Each tooth was firmly seated in a dedicated holding device (ProTrain; Simit Dental, Mantova, Italy) containing the accompanying conductive gel. One electrode of the apex locator was connected to the base of the ProTrain to complete the conductive circuit. The teeth were allocated equally to two groups and prepared with a continuous single-file system (One Curve; Micro-Mega, Besançon, France) driven by one of two integrated endodontic motor/EAL systems: Group 1 (Connect Drive; VDW, Munich, Germany) and Group 2 (E-Connect S; Eighteeth, Jiangsu, China). Instrumentation continued until the “apex” signal was displayed on the iPad or handpiece screen; this signal represents the auto-apical-reverse endpoint of each integrated system, which is intended to indicate the major apical foramen. The correspondence between this electronic signal and the anatomical major apical foramen was assumed on the basis of the manufacturers' operating specifications and was not independently validated in the present study. The conductive gel (ProTrain, Simit Dental Srl, Italy), 5.25% NaOCl irrigant solution (Cerkamed, Poland) for irrigation, new file per tooth were used for repeatability of the protocol. Each tooth was then reinserted into its original putty index for the second CBCT scan. Each tooth was then reinserted into its original putty index for the second CBCT scan.

### 3D reconstruction and endpoint measurement

Three-dimensional reconstruction was performed using Mimics Innovation Suite (Materialise, Leuven, Belgium). Thresholding and scan-registration functions were used to isolate the canal system and to superimpose the pre- and post-preparation images, which were assigned different colors and superimposed to detect the preparation endpoint. The shortest distance from this endpoint to the major apical foramen was then measured and recorded. CBCT measurements were repeated, and intra- and inter-examiner reliability were quantified using the intra-class correlation coefficient.

### Outcome measures and abbreviations

To improve clarity, the outcome variables are defined here before they appear in the Results. Actual length (AL) is the directly measured reference length from the occlusal reference to the major apical foramen. Actual auto-apical-reverse length (AAARL) is the file length recorded when the instrument triggered the auto-reverse signal during dynamic instrumentation. Root canal length measured on the computer (RCL3D) is the pre-preparation canal length obtained from the 3D reconstruction. Auto-apical-reverse length measured on the computer (AARL3D) is the post-preparation endpoint-to-foramen distance obtained from the 3D reconstruction. The difference variables in Table 1 are defined as follows: ACD=AL minus AAARL for the Connect Drive motor; 3DCD = RCL3D minus AARL3D for the Connect Drive motor; AECS=AL minus AAARL for the E-Connect S motor; and 3DECS = RCL3D minus AARL3D for the E-Connect S motor. All values are expressed in millimeters.

**Table 1:** Distribution (minimum, quartiles, median, maximum, in millimetres) of the actual-vs.-measured length differences for the two systems, with the between-device comparison.

Variable	Minimum	25th pct	Median	75th pct	Maximum	*p*-value
ACD	−0.55	−0.283	0	0	0	0.387^†^
3DCD	−0.43	−0.275	−0.07	0	0	
AECS	−0.79	−0.243	−0.11	0	0	
3DECS	−0.76	−0.383	−0.14	0	0	

ACD: AL minus AAARL (Connect Drive); 3DCD: RCL3D minus AARL3D (Connect Drive); AECS: AL minus AAARL (E-Connect S); 3DECS: RCL3D minus AARL3D (E-Connect S); pct: percentile.

† Between-device comparison. All values in millimeters. All maximum values are 0 and all minimum values are negative. No positive (long) deviations were recorded using the present measurement method; the deviations therefore ranged from zero (at the major apical foramen) to a maximum of 0.79 mm short of it. However, over-extension beyond the major apical foramen could not be quantitatively assessed by the 3D method, and the absence of recorded positive deviations should not be interpreted as definitive evidence that over-extension never occurred.

### Statistical analysis

Data were analyzed in MedCalc (MedCalc Software, Ostend, Belgium) with significance set at 0.05. Normality of the difference variables was assessed before parametric testing. Agreement between each measured length and the actual length was evaluated using Bland–Altman analysis, reporting the mean bias, the standard deviation of the differences, and the 95% limits of agreement with their confidence intervals; proportional bias was tested by regressing the differences on the means (12, 13). The presence of a fixed bias was assessed from the 95% confidence interval of the mean bias. Clinical accuracy was additionally interpreted against the conventional tolerance thresholds of ±0.5 mm and ±1.0 mm. Because the two motors were tested on independent groups of teeth, between-device differences were analyzed using a method appropriate for independent samples (Mann–Whitney *U*-test or the independent-samples equivalent) rather than a related-samples test. A non-significant within-device comparison was interpreted as the absence of a detectable systematic difference and not as proof of equivalence or agreement.

## Results

The root canal lengths measured by the two integrated motor/EAL systems are summarized in Tables 2, 3. For each device, the mean bias and its 95% confidence interval describe the systematic deviation from the actual length; a fixed bias was inferred when the confidence interval excluded zero, and proportional bias was assessed by linear regression of the differences on the means.

**Table 2:** Bland–altman mean bias (mm), 95% confidence interval, paired-comparison and regression *p*-values, and presence of fixed or proportional bias for the connect drive (VDW) system relative to the actual root canal length.

Comparison	Mean bias (95% CI)	*p* (paired/regression)	Fixed bias	Proportional bias
AL–AAARL	0.1323 (0.04059 to 0.2240)	0.0085*/0.699	Yes	No
AL–RCL3D	0.08615 (−0.09256 to 0.2649)	0.3142/0.8276	No	No
AL–AARL3D	0.24 (0.03196 to 0.4480)	0.0272*/0.8368	Yes	No
AARL–RCL3D	−0.1538 (−0.2722 to −0.03549)	0.0151*/0.8272	Yes	No
AAARL–AARL3D	0.1077 (−0.1276 to 0.3430)	0.3383/0.759	No	No
RCL3D–AARL3D	0.04615 (−0.1802 to 0.2725)	0.6647/0.8368	No	No

AL, actual length; AAARL, actual auto-apical-reverse length; RCL3D, root canal length measured on the computer; AARL3D, auto-apical-reverse length measured on the computer. *p < 0.05. All biases are in millimeters.

**Table 3:** Bland–altman mean bias (mm), 95% confidence interval, paired-comparison and regression *p*-values, and presence of fixed or proportional bias for the E-connect S system relative to the actual root canal length.

Comparison	Mean bias (95% CI)	*p* (paired/regression)	Fixed bias	Proportional bias
AL–AAARL	0.2354 (0.08312 to 0.3877)	0.0056*/0.5647	Yes	No
AL–RCL3D	0.09154 (−0.07868 to 0.2618)	0.2641/0.8914	No	No
AL–AARL3D	0.2623 (0.03135 to 0.4933)	0.0292*/0.8593	Yes	No
AAARL–RCL3D	−0.1708 (−0.3169 to −0.02460)	0.0257*/0.8771	Yes	No
AAARL–AARL3D	0.02692 (−0.1834 to 0.2372)	0.785/0.7087	No	No
RCL3D–AARL3D	0.1438 (−0.09410 to 0.3818)	0.2124/0.6702	No	No

Abbreviations as in Table 1. *p < 0.05. All biases are in millimeters.

With the Connect Drive system, the pre-preparation computed length (RCL3D) showed no fixed bias relative to the actual length (mean bias 0.086 mm; 95% CI −0.093 to 0.265), whereas the dynamic auto-reverse endpoints (AAARL and AARL3D) showed small but statistically detectable fixed biases (0.13–0.24 mm), indicating that instrumentation tended to stop slightly short of the actual length. No proportional bias was detected for any comparison.

The E-Connect S system showed the same pattern: no fixed bias for the pre-preparation computed length (RCL3D; mean bias 0.092 mm; 95% CI −0.079 to 0.262), and small fixed biases for the dynamic auto-reverse endpoints (0.24–0.26 mm). No proportional bias was detected. In both systems the magnitude of the mean bias was well below the conventional ±0.5 mm clinical tolerance, although the confidence intervals approached it.

No statistically significant difference was detected between the two systems for either the directly measured deviation or the 3D-derived deviation (*p* = 0.387). Both systems tended to stop at or slightly short of the major apical foramen, with all recorded deviations within 0.8 mm of the reference.

## Discussion

The present study evaluated the accuracy of two integrated endodontic motor/EAL systems for dynamic working length determination using a CBCT-based three-dimensional subtraction protocol. Neither system reproduced the actual length exactly: small but statistically detectable fixed biases were present, with the instrument tending to stop at or slightly short of the major apical foramen. The first null hypothesis was rejected because fixed biases were present, whereas the second null hypothesis was not rejected, because no significant difference was detected between the two systems.

These findings are consistent with previous reports on integrated motor/EAL systems. Cruz et al. found that two integrated rotary motors with apex-locating function measured working length within clinically acceptable limits in single-rooted teeth (6), and Borges et al. similarly reported that an integrated rotary motor/EAL could not determine the canal length exactly, with fixed biases comparable to those observed here, and recommended further validation (11). A recent systematic review of *in vitro* studies concluded that integrated EALs are generally reliable but that the literature is heterogeneous in protocol and analysis and frequently at unclear risk of bias (17), reinforcing the need for standardized reference standards and agreement statistics such as those used in the present study.

Recent *ex vivo* evidence on contemporary stand-alone and multifunctional locators provides additional context. Ayhan and Aykanat reported comparable accuracy among the X-Smart Pro+, Root ZX Mini, and Propex Pixi in determining the apical constriction, with no significant inter-device difference and mean deviations below 0.25 mm (23). Teja et al. showed that integrated endomotors (EnDrive and X-Smart Pro+) remained within clinically acceptable limits (−0.15 to −0.50 mm) across saline, sodium hypochlorite, and EDTA irrigants (24), and Bailey et al. found that integrated handpieces (E-Connect S + and Tri Auto ZX2+) placed 84%–94% of measurements within ±0.5 mm and essentially all within ±1.0 mm of the actual length (25). The small fixed biases observed in the present study fall within this same clinically acceptable range, supporting the view that integrated systems perform comparably to recently evaluated stand-alone devices while adding real-time, canal-specific control during shaping (26).

The CBCT-based reference workflow offers an effective and non-invasive method for investigating the endpoint of endodontic preparation. The repeatability of measurement was supported by selecting the shortest distance between the two determined points. A principal limitation is that the 3D reconstruction cannot detect the instrument tip when it passes beyond the major apical foramen; consequently, the distance of any over-instrumentation could not be quantified and was recorded only as a categorical sign. Because over-extension is precisely the clinical risk associated with auto-reverse systems, the present design can characterize the detectable post-instrumentation endpoint under the selected conditions but cannot fully assess over-instrumentation; broad claims of exact accuracy are therefore avoided.

CBCT is a high-value surrogate rather than a histologic gold standard. A systematic review reported that preoperative CBCT identified the apical foramen on average 0.40 mm coronal to its true position (mean absolute difference 0.48 mm) (14), consistent with the 2025 AAE/AAOMR update emphasizing indication-based, task-specific imaging rather than routine exposure for working-length measurement alone (15). CBCT acquisition parameters — voxel size, slice thickness, field of view, metal status, and artefact-reduction settings — are central determinants of apparent device performance (3, 4, 16, 18–22) and are reported here accordingly.

Mandibular single-canal premolars were appropriate specimens because they are commonly extracted for orthodontic reasons and present a relatively reproducible single-canal anatomy. The diameter of the apical foramen and the apical third are important because they can affect both electronic measurement and the detectability of dimensional change on the CBCT.

Clinically, the integrated EAL provides real-time confirmation during shaping, but the working length should be confirmed in combination with radiographs and not determined solely by the auto-reverse endpoint (6). This is consistent with our finding of small fixed biases: although the average deviation was well within ±0.5 mm, the confidence intervals approached this threshold, so independent confirmation remains prudent.

This study has several limitations. Only two devices, one file system, one tooth type, and 26 extracted teeth were evaluated, so the findings cannot be generalized to all integrated systems. The *in vitro* model cannot reproduce the periodontal and periapical environment; the 3D method cannot quantify over-instrumentation, and the sample-size estimation used an alpha of 0.10 and was powered for the within-device comparison. Future studies should use larger samples, clinically relevant tolerance thresholds (±0.5 and ±1.0 mm), standardized reference methods, and prospective paired designs.

## Conclusion

Under the present *in vitro* conditions, neither tested integrated endodontic motor/EAL system (Connect Drive and E-Connect S) produced measurements identical to the directly measured reference length; both showed small fixed biases and tended to stop at or slightly short of the major apical foramen. No significant difference was observed between the two systems. Because the deviations were small but the confidence intervals approached the ±0.5 mm clinical tolerance, the working length should be confirmed with complementary methods rather than relying solely on the auto-reverse endpoint. Further studies using larger samples, clinically relevant tolerance thresholds, and standardized reference methods are warranted.

## Data Availability

The raw data supporting the conclusions of this article will be made available by the authors, without undue reservation.

## References

[1] Nguyen PN Pham KV. Endodontic length measurements using different modalities: an *in vitro* study. J Int Soc Prev Community Dent. (2020) 10(6):752–8. 10.4103/jispcd.JISPCD_357_20 33437709 PMC7791584

[2] Pham K. Endodontic length measurements using 3D endo, cone-beam computed tomography, and electronic apex locator. BMC Oral Health. (2021):21:271. 10.1186/s12903-021-01625-w 34006262 PMC8130300

[3] Pham V-K Pham T-L-K. Root canal length estimated by cone-beam computed tomography at different slice thicknesses, dedicated endodontic software, or measured by an electronic apex locator. Sci Rep. (2022) 12(1):6531. 10.1038/s41598-022-10534-z 35444163 PMC9021240

[4] Van Pham K. Endodontic length measurements using cone beam computed tomography with dedicated or conventional software at different voxel sizes. Sci Rep. (2021) 11(1):9432. 10.1038/s41598-021-88980-4 33941828 PMC8093273

[5] Van Pham K Kim Khuc N. The accuracy of endodontic length measurement using cone-beam computed tomography in comparison with electronic apex locators. Iran Endod J. (2020) 15(1):12–7. 10.22037/iej.v15i1.26720 36704319 PMC9723209

[6] Cruz ATG Wichnieski C Carneiro E Da Silva Neto UX Gambarini G Piasecki L. Accuracy of 2 endodontic rotary motors with integrated apex locator. J Endod. (2017) 43(10):1716–9. 10.1016/j.joen.2017.05.012 28735795

[7] Connert T Judenhofer MS Hülber JM Schell S Mannheim JG Pichler BJ. Evaluation of the accuracy of nine electronic apex locators by using Micro-CT. Int Endod J. (2018) 51(2):223–32. 10.1111/iej.12814 28675449

[8] Beach DA. CBCT Use in endodontic diagnosis. Dent Today. (2016) 35(2):80. 82-83. PMID: 26995838. 26995838

[9] Vasconcelos BC Bastos LM Oliveira AS Bernardes RA Duarte MA Vivacqua-Gomes N. Changes in root canal length determined during mechanical preparation stages and their relationship with the accuracy of root ZX II. J Endod. (2016) 42(11):1683–6. 10.1016/j.joen.2016.07.022 27616540

[10] Wolgin M Grundmann MJ Tchorz JP Frank W Kielbassa AM. *Ex vivo* investigation on the postoperative integrity of the apical constriction after the sole use of electronic working length determination. J Dent. (2017) 64:52–7. 10.1016/j.jdent.2017.06.005 28642058

[11] Borges JHSM De Carvalho FB Cangussu MCT Peters OA Bahia MGA Pereira ÉSJ. Accuracy of an endodontic rotary motor with integrated apex locator to determine the root canal length. Arq Odontol. (2023) 59:e10. (p. 106-113). 10.35699/2178-1990.2023.45739

[12] Bland JM Altman DG. Statistical methods for assessing agreement between two methods of clinical measurement. Lancet. (1986) 1(8476):307–10. 10.1016/S0140-6736(86)90837-8 2868172

[13] Bland JM Altman DG. Measuring agreement in method comparison studies. Stat Methods Med Res. (1999) 8(2):135–60. 10.1177/096228029900800204 10501650

[14] Paterson A Franco V Patel S Foschi F. Use of preoperative cone-beam computed tomography to aid in establishment of endodontic working length: a systematic review and meta-analysis. Imaging Sci Dent. (2020) 50(3):183–92. 10.5624/isd.2020.50.3.183 33005575 PMC7506090

[15] Sousa Melo SL Fayad MI Gohel A Johnson BR Kalathingal S Mahdian M. AAE And AAOMR joint position statement: use of cone-beam computed tomography in endodontics 2025 update. J Endod. (2026) 52(1):4–13. 10.1016/j.joen.2025.09.008 41412684

[16] Koç S Harorlı H Kuştarcı A. Comparative accuracy of cone-beam computed tomography and integrated electronic apex locator in apically resected teeth: an *ex vivo* study. Odontology. (2026) 114(2):844–51. 10.1007/s10266-025-01166-6 40760289

[17] La Rosa GRM Angjelova A Jovanova E Apostolska S Santos JM Pedullà E. Integrated electronic apex locators for determining working length: a systematic review of *in-vitro* studies. Odontology. (2025) 113(3):918–29. 10.1007/s10266-024-01039-4 39661212

[18] Koç S Harorlı H Kuştarcı A. Comparative evaluation of the accuracy of electronic apex locators and cone-beam computed tomography in detection of root canal perforation and working length during endodontic retreatment. BMC Oral Health. (2024) 24(1):953. 10.1186/s12903-024-04713-9 39152371 PMC11328487

[19] Izadi A Golmakani F Kazeminejad E Mahdavi Asl A. Accuracy of working length measurement using cone beam computed tomography at three field of view settings, conventional radiography, and electronic apex locator: an ex-vivo study. Eur Endod J. (2024) 9(3):266–72. 10.14744/eej.2023.97769 39102662 PMC11413608

[20] Pinto JC De Faria Vasconcelos K Leite AF Wanderley VA Pauwels R Oliveira ML. Image quality for visualization of cracks and fine endodontic structures using 10 CBCT devices with various scanning protocols and artefact conditions. Sci Rep. (2023) 13(1):4001. 10.1038/s41598-023-31099-5 36899046 PMC10006407

[21] Freitas A Peixoto LR Mariz Suassuna FC Bento PM Maia Amorim AMA Rovaris Silva K. The effects of different metal posts, cements, and exposure parameters on cone-beam computed tomography artifacts. Imaging Sci Dent. (2023) 53(2):127–35. 10.5624/isd.20220185 37405202 PMC10315228

[22] Alves LVGL Pires CRF Sousa-Neto MD Prado HS Mazzi-Chaves JF Candemil AP. Metal artifact reduction tool and mA levels impact on the diagnosis of fracture extension in endodontically treated teeth using cone-beam CT. Clin Oral Investig. (2024) 28(10):531. 10.1007/s00784-024-05945-3 39298025

[23] Ayhan M Aykanat B. *Ex vivo* accuracy of three electronic root canal length measurement devices: x-smart pro+, root ZX mini, and propex pixi. BMC Oral Health. (2025) 25(1):1072. 10.1186/s12903-025-06482-5 40604896 PMC12224375

[24] Teja KV Vasundhara KA Solete P Rossi-Fedele G. Accuracy of apex locator integrated endomotors in estimating working length in the presence of endodontic irrigants: an *ex vivo* study. Aust Endod J. (2025) 51(2):555–62. 10.1111/aej.70002 40772583 PMC12712882

[25] Bailey D Anderson R Brady K Kwon P Browne D Amaral RR. An ex-vivo study comparing the accuracy of the E-connect S+ and morita tri auto ZX2 + endodontic handpieces in root canal length determination. J Endod. (2024) 50(7):1004–10. 10.1016/j.joen.2024.04.007 38631475

[26] Pisano M Sangiovanni G Frucci E Scorziello M Benedetto GD Iandolo A. Evaluation of the accuracy of electronic apex locators in modern endodontics: an umbrella review. Medicina (Kaunas). (2024) 60(10):1709. 10.3390/medicina60101709 39459496 PMC11510102

